# Development of a high-throughput field phenotyping rover optimized for size-limited breeding fields as open-source hardware

**DOI:** 10.1270/jsbbs.21059

**Published:** 2022-02-08

**Authors:** Ken Kuroki, Kai Yan, Hiroyoshi Iwata, Kentaro K. Shimizu, Toshiaki Tameshige, Shuhei Nasuda, Wei Guo

**Affiliations:** 1 Graduate School of Agriculture, Kyoto University, Kitashirakawaoiwake-cho, Sakyo, Kyoto 606-8502, Japan; 2 Graduate School of Science, The University of Tokyo, 7-3-1 Hongo, Bunkyo, Tokyo 113-0033, Japan; 3 LabRomance Inc, 1-3-29-2F Ureshino, Fujimino, Saitama 356-0056, Japan; 4 Graduate School of Agricultural and Life Sciences, The University of Tokyo, 1-1-1 Yayoi, Bunkyo, Tokyo 113-8657; 5 Department of Evolutionary Biology and Environmental Studies, University of Zurich, Zurich 8057, Switzerland; 6 Kihara Institute for Biological Research, Yokohama City University, 641-12 Maioka, Totsuka, Yokohama, Kanagawa 244-0813, Japan; 7 Department of Biology, Faculty of Science, Niigata University, 8050 Ikarashi 2-no-cho, Nishi, Niigata 950-2181, Japan; 8 Graduate School of Agricultural and Life Sciences, The University of Tokyo, 1-1-1 Midori, Nishitokyo, Tokyo 188-0002, Japan

**Keywords:** high-throughput phenotyping, open-source hardware, field phenotyping rover, image processing, proximal sensing

## Abstract

Phenotyping is a critical process in plant breeding, especially when there is an increasing demand for streamlining a selection process in a breeding program. Since manual phenotyping has limited efficiency, high-throughput phenotyping methods are recently popularized owing to progress in sensor and image processing technologies. However, in a size-limited breeding field, which is common in Japan and other Asian countries, it is challenging to introduce large machinery in the field or fly unmanned aerial vehicles over the field. In this study, we developed a ground-based high-throughput field phenotyping rover that could be easily introduced to a field regardless of the scale and location of the field even without special facilities. We also made the field rover open-source hardware, making its system available to public for easy modification, so that anyone can build one for their own use at a low cost. The trial run of the field rover revealed that it allowed the collection of detailed remote-sensing images of plants and quantitative analyses based on the images. The results suggest that the field rover developed in this study could allow efficient phenotyping of plants especially in a small breeding field.

## Introduction

High throughput field phenotyping is a key technology for accelerating plant science, both in basic science for large-scale genetic and physiological research projects and in agricultural application such as streamlining selection processes in breeding programs (Araus and Cairns 2014). In recent years, the importance has rapidly increased in response to changes in the systems surrounding agriculture, such as increasing food demand, climate change, and the spread of organic farming. Conventional manual phenotyping methods are generally labor-intensive or costly due to their inherently limited efficiency. This limitation in the efficiency of phenotyping has become a major bottleneck of genome-phenotype association methods, such as Genome-Wide Association Studies and Genomic Selection, and resulted in a strong demand for more efficient phenotyping methods (Furbank and Tester 2011). In addition, manual phenotyping can also be inaccurate because it relies on subjective assessments by human (Li *et al.* 2014).

To increase the efficiency as well as accuracy in phenotyping, researchers have developed various high-throughput phenotyping methods. The development has been taking advantage of the rapid progress in digital technologies both in terms of hardware and software (Chen *et al.* 2014). Systems with belt conveyer were developed for indoor environment cultivations to automate and accelerate phenotyping (Fujita *et al.* 2018, Tisné *et al.* 2013). Alternatively, some other systems use many static cameras for phenotyping (Lee *et al.* 2018, Tausen *et al.* 2020). For cultivations in outdoor environments, where introducing such a complex system is challenging, large gantry cranes equipped with various sensors have been deployed (Virlet *et al.* 2016). Another prominent phenotyping approach is remote sensing with unmanned aerial vehicles (UAVs), which offer efficient and affordable high-throughput phenotyping and already have numerous applications (Fukano *et al.* 2021, Watanabe *et al.* 2017, Xie and Yang 2020, Yang *et al.* 2017). UAVs, however, have limited battery duration and payload, restricting the amount and variety of sensors that can be equipped, they are also subject to a trade-off between obtaining high-resolution images by lowering altitude and avoiding their downwash effect on plants (Yang *et al.* 2020). UAV operations are also subject to unpredictable weather conditions (Thibbotuwawa *et al.* 2020) and local regulations especially in populated areas (Stöcker *et al.* 2017).

Thus, these restrictions on high-throughput phenotyping methods suggest that breeding fields with limited scale and nearby urban areas, typical conditions for countries in regions such as Asia, ground facilities and UAVs offer only partial solutions for phenotyping bottleneck. In this study, we report the development and operation trial of a lightweight ground-based field phenotyping rover. Our field rover was designed to conduct high-throughput phenotyping and to be easy to introduce and operate even in size-limited fields and in urbanized environments where UAVs and large ground facilities cannot be introduced. Moreover, according to open hardware principle, an analogous concept to open-source software, we make its design and detailed specs openly available and free to use at our GitHub repository, so that readers can assemble it on their own (Fisher *et al.* 2015, Powell 2012).

## Materials and Methods

Inspired by a previous study (Thompson *et al.* 2018) in aspects such as the general frame design, camera layout, usage of a transmitter for radio-controlled aircrafts, we developed a ground-based field phenotyping rover. Notable differences of our system from the previous one are: (1) we integrated a compact and powerful computer as the “brain” of our field rover, enabling implementation of various real-time image processing including the following (2) we made our field rover semi-automatic with the computational ability of the computer, which can recognize fiducial markers on the ground and follow the correct course, (3) we aimed to achieve a high agility to enter, leave, and move between fields overcoming hurdles with portable slopes, given that experimental fields in Japan and other Asian countries are, unlike those in Western countries, often surrounded by ridges and structures with some height, such as waterways in a rice paddy field, and (4) we used commercially available batteries for UAVs, because they are superior to naked lithium-ion batteries in terms of safety and ease of use and maintenance. The components are shown in Table 1.

### General design

**Fig. 1.**
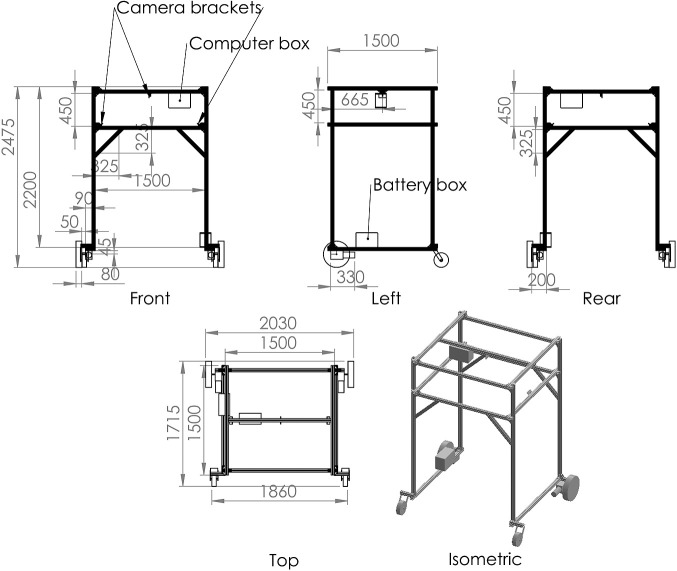
The drawing for our field phenotyping rover by the 3D CAD software SolidWorks (Dassault Systemes SE, Vélizy-Villacoublay, France). The 45-mm^2^ aluminum frame was assembled and equipped with components for control and driving. The CAD data file is available our GitHub repository.

**Fig. 2.**
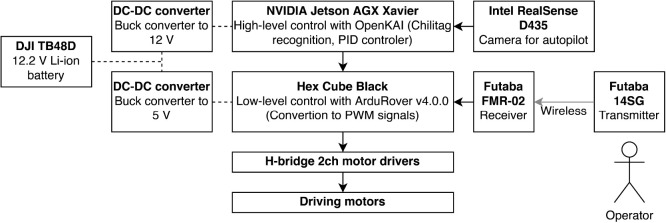
The hardware and software scheme.

The chassis is an inverted U-shaped, approximately 1.5 m long, 1.5 m wide, and 2.5 m high, with internal vertical clearance of 2.0 m, allowing it to pass over tall plants. We used aluminum frames (MISUMI Group Inc., Tokyo, Japan) for ease of assembly and modification of the body. The body was then equipped with driving wheels connected to the driving motors, trailing swivel casters, and plastic boxes containing electric components and camera mounts. The total weight is approximately 70 kg. In addition, we added opaque white cover films (C.I. Takiron Corp., Tokyo, Japan) to mitigate the high contrast caused by direct sunlight, which can preclude the measurement of plant phenotypes from captured images. Based on our design, the frame geometry can easily be changed for different purposes and requirements of phenotyping because welding is not necessary to assemble or modify the field rover. The design drawing is shown in Fig. 1. The overall control hardware and software diagram is shown in Fig. 2.

### Driving module

The field rover is four-wheeled, with two driving and two trailing and free-rotating casters. It is turned by controlling motors connected to each driving wheel differentially. Pivot turns are also possible, making it suitable for operation in a field with limited size. The front and rear tires are approximately 350 mm diameter and 85 mm wide, and 210 mm diameter and 60 mm wide, respectively.

### Power supply

We used batteries for industrial unmanned aerial vehicles, DJI TB48D (22.2 V, 5,700 mAh; SZ DJI Technology Co., Shenzhen, China). The batteries are easy to purchase and easy to use because of built-in protection circuits and battery indicators. The field rover uses two or three of the batteries: one for powering electric components and the other or two for the driving module. One driving battery can power the motors approximately for a one-hour long phenotyping operation and seems sufficient in general practical applications. For additional safety, we added an emergency button on the circuit so that it can cut off the entire power supply in case of emergency.

### Control software

For high-level driving control, we used an open-source software OpenKAI (https://github.com/yankailab/OpenKAI), which is a high-level vehicle control software implemented in C++ language. It can receive real-time images from the navigation camera, detect 2D fiducial markers in the images, and control steering depending on the marker location and angle relative to the camera frame. Specifically, it automatically recognizes Chilitag (Bonnard *et al.* 2013); Chilitag Technology Co., Ltd., Kaohsiung city, Taiwan), a fiducial marker system used similarly in other studies in robotics (Tan *et al.* 2016, Tsoy *et al.* 2019). The software library for marker recognition used with OpenKAI in this study is available as open-source software (https://github.com/chili-epfl/chilitags). The steering is controlled based on the recognition result so that the marker is located on the center horizontally and angled straight by a Proportional-Integral-Derivative (PID) controller. PID controller controls vehicles by combining the proportional term, integral term, and derivative term, calculated by the deviation measured at the current moment, its cumulative sum over a certain period, and its rate of change, respectively. The amount of steering control is then added to the constant speed driving either to go forward or backward by autopilot, so that the eventual driving and turning speed can be obtained.

Because we control steering to counteract both position and angular deviation of the marker, we have two PID controllers, one for position and the other for angle, and linearly combine the two. We found the derivative term helps stabilization only for the angular deviation and omitted it for the position deviation because of its slower and more stable change than that of the angular deviation, effectively making it a PI controller. The resulting equations are as follows:

$$\begin{array}{lcl} u^{\text{position}}\left(t\right) & = & K_{p}^{\text{position}}e^{\text{position}}\left(t\right)\\ & & +K_{i}^{\text{position}}\int_{t-t_{1}^{\text{position}}}^{t}e^{\text{position}}\left(\tau \right)d\tau \end{array}$$ (1)

$$\begin{array}{lcl} u^{\text{angle}}\left(t\right) & = & K_{p}^{\text{angle}}e^{\text{angle}}\left(t\right)\\ & & +K_{i}^{angle}\int_{t-t_{1}^{\text{angle}}}^{t}e^{\text{angle}}\left(\tau \right)d\tau +K_{d}^{\text{angle}}\frac{\text{de}(t)}{\text{dt}} \end{array}$$ (2)

$$u^{\text{total}}\left(t\right)=u^{\text{position}}\left(t\right)+u^{\text{angle}}\left(t\right)$$ (3)

where *e* denotes the deviation of the fiduciary marker in either the position (*e*^position^) or the angle (*e*^angle^) at the current frame. *K*_p_, *K*_i_, *K*_d_ are scaling constants for the proportional, integral, and differential terms of the deviation, respectively. The intervals of integral are determined by $t_{1}^{\text{position}}$ and $t_{1}^{\mathrm{angle}}$ , also to be set separately for position and angular deviations, respectively. In the presented setup all the variables in the above equations are given in camera screen coordinate unit (pixel coordinate normalized by screen width and height) as the navigation camera is mounted on a known fixed height on the chassis. The central region of the camera screen used for control is much smaller compared to the entire field of view, in such a setup the camera intrinsic parameters play a limited role in the position and angle detections, thus we omitted them for the sake of simplicity. The configuration file for OpenKAI including the PID controller parameters is available at our GitHub repository.

This high-level output is then transmitted to the low-level controlling software ArduRover v4.0.0 (https://github.com/ArduPilot/ardupilot), a component of ArduPilot, an open-source autopilot software system for unmanned vehicles. ArduRover then converts the driving speed input to a low-level electric signal using the Pulse-Width Modulation (PWM) method for each motor. PWM switching controls motor current on and off as pulse signal within a short interval, and the duration of each pulse determines the amount of drive of the motor.

### Hardware

As the main on-board computer, we used Jetson AGX Xavier (NVIDIA Corp., CA, USA), which was connected to a USB camera RealSense D435 (Intel Corp., CA, USA) mounted downward in front of the field rover with a clamp mount (SmallRig, Guangdong, China) to capture fiducial markers placed on the ground to guide the field rover along furrows. The main computer runs OpenKAI to calculate the necessary amount of speed and steering control. The signal is then sent to and received by Hex Cube Black controller (ProfiCNC, VIC, Australia), an implementation of Pixhawk standard, multi-purpose drone controller for unmanned vehicles. The controller then outputs PWM signals calculated with ArduRover and sends the signals to the motor controller boards, which directly control the larger electric current for the motors. The rating output of a motor is 300 Watt.

**Fig. 3.**
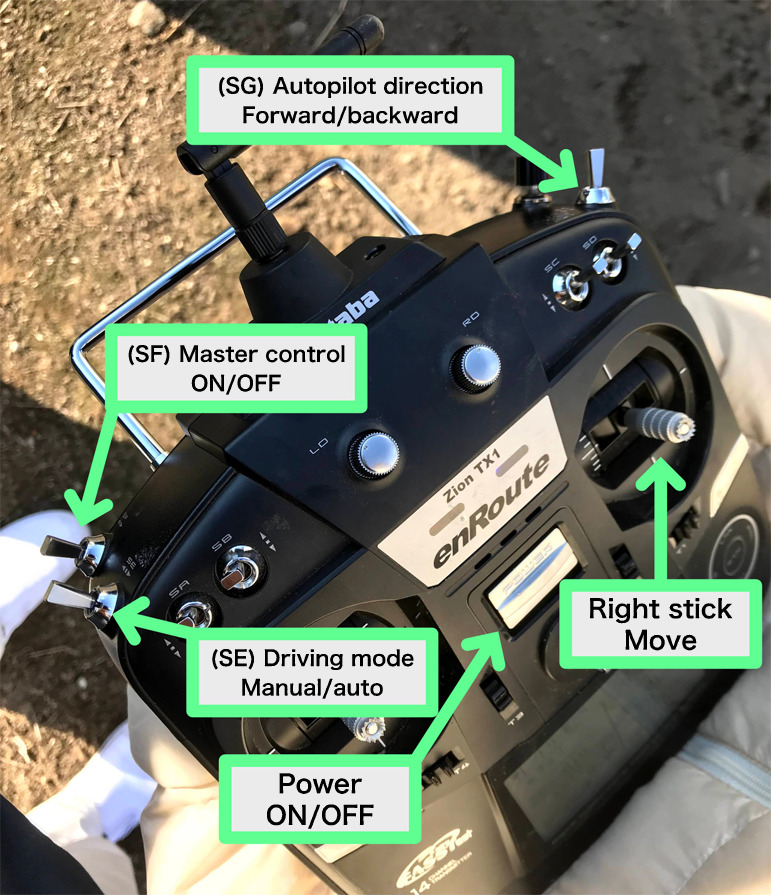
Transmitter configurations.

We used 14SG (Futaba Corp., Chiba, Japan) as a remote-control transmitter. It was designed as a transmitter for aerial vehicles and had a sufficient range for our purpose, offering a stable radio connection. The transmitter was remotely linked to a receiver FMR-02 (Futaba Corp.) connected to Hex Cube Black. We setup the transmitter such that the right stick drives the field rover, the left-back switch (SF) is the master control, the left-front switch (SE) selects auto/manual modes, and the right-front switch (SG) reverses the driving direction for the autopilot (Fig. 3).

Pre-generated image files of Chilitag fiduciary markers were obtained from its GitHub repository (https://github.com/chili-epfl/chilitags#tag-generation). We chose big-border version for our purpose, and Pronate Inc. (Tokyo, Japan) printed them on 8-cm^2^ aluminum boards. We then pierced holes on their corners and fixed them on the field with metal pegs.

### Wheat cultivation for trial operation

We developed the field phenotyping rover and operated it at Kyoto Farm, Graduate School of Agriculture, Kyoto University, Japan (N35.0325, E135.7828) during the winter wheat cultivation season 2020 to 2021. The wheat was cultivated on furrows approximately 24 m long and 1 m wide. Wheat cultivars of diverse genotypes were densely planted, each of them on a cell tray 25 cm long, with a 10-cm gap on each end between the adjacent plots. Each plot contained 10 individual plants unless they failed to germinate. An 80-cm margin was left on each side of furrows to keep enough clearance for the passage of the field rover. We put anti-weed sheet on the field to prevent weed and muddy conditions.

### Image acquisition

We used three RX0 II digital cameras (Sony Corp., Tokyo, Japan), mounted on right, top, and left of the chassis, all directed downward pointing to the center of the vehicle. This layout of cameras was intended to allow 3D reconstruction of the field by combining both nadir and oblique images using structure from motion (SfM) software. The cameras have an interval shutter function with a minimum interval of one second.

For our trial operation, we used the manual (M) mode for the cameras and set the shutter speed to 1/640 s to avoid motion blur due to vehicle movement as well as plant movement caused by wind. Aperture was set to F4 and ISO sensitivity was set auto to allow adjustment for illumination conditions due to varying weather and time. We used Extreme Pro V30 micro-SD card (SanDisk LLC, CA, USA) to save images of each camera. It guarantees the writing speed of 30 MB/s as a Video Speed Class 30 card, which is sufficient for a one second interval shutter with each image approximately 10 MB large.

### Image preprocessing

For each day, images capturing one furrow were grouped together as an image set. Each set of images was then used as an input for structure-from-motion software PIX4Dmapper (version 4.6.4; PIX4D S.A., Prilly, Switzerland) after cropping margins to remove the cart chassis. The output of PIX4Dmapper along was then given to EasyIDP (Wang *et al.* 2021) to extract each plot from the source images. We predefined geocoordinates for each plot and used them for plot extraction. For quantitative analyses, we used 512*512-pixel region around the center of each image captured by the top camera. Although one plot is captured in multiple images, we selected images close to the nadir angle to minimize image distortion.

### Image analysis using deep learning

We used YOLOv5 (https://github.com/ultralytics/yolov5) to detect heads and generates a bounding box for each of them. For this purpose, a total of 75 plots of 39 cultivars from the same population were grown in 2020 and 3,514 images were obtained by a hand-held camera G900SE (Ricoh Company, Ltd., Tokyo, Japan). Plots were split to two furrows of approximately the same length, and images from one furrow were used for training, the other for validation. Among several versions of YOLOv5, we used YOLOv5m, a medium-sized model, to balance model flexibility and computational performance, as well as large size of mini batch. Computation was conducted on a workstation using one Tesla P40 graphic processing unit (NVIDIA Corp.). The number of wheat heads were analyzed as a time-series and the heading date was defined as when the number of detected heads surpassed 50% of the maximum number of the plot.

## Results

We conducted a phenotyping operation using the field rover as follows. It departs its depot, goes into the field, runs approximately at 1 km/h over the entire length of a furrow and then goes back the same path before switching to the next furrow, before going back to the depot having captured all of the designated furrows. We placed 8-cm^2^ fiduciary markers 1 m apart from each other, a distance we found necessary for stable semi-automatic driving over the straight line due to minor surface irregularities on the ground. Because automatic driving of the field rover from one furrow to another remained challenging due to the space limitation of the field, for the current version, it requires a manual operation to switch the field rover from one furrow to another. Note that even when manually driving, all of our operators with varying experience levels were able to follow an appropriate driving track with stable speed without trouble.

**Fig. 4.**
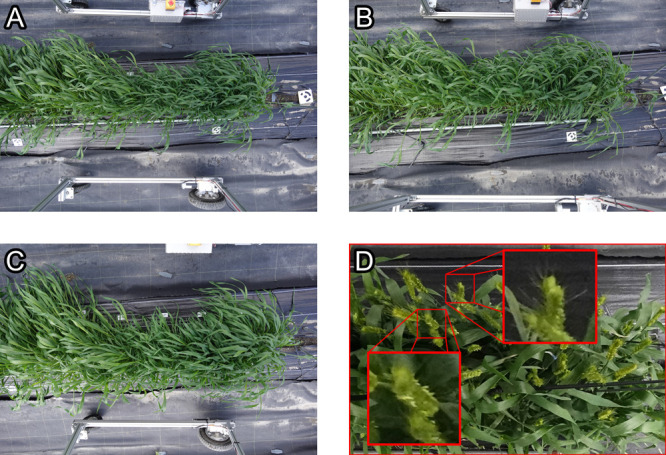
Images captured by our field phenotyping rover on the field trial. (A–C) Images from center, right, and left camera, respectively. Six different cultivars each consisted of 10 individual plants are observed. Photo was taken on April 5, 2021. (D) An image from the top camera taken at the same location as (A–C), but in the heading season. Wheat spikes, as well as flowers (bottom) and awns (right) when magnified, are clearly visible. Photo was taken on April 30, 2021 and cropped from the original image for the purpose of visibility here.

**Fig. 5.**
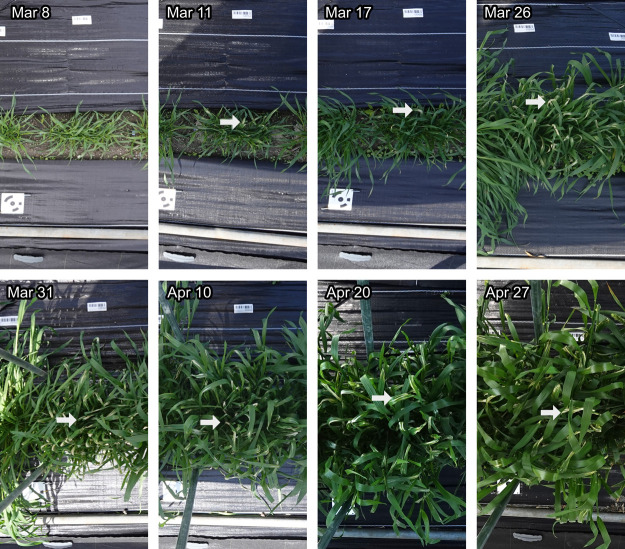
Follow-up and retrospective observation of physiological disorder. All of the images are taken approximately at the same location over a time series. The disorder was noticed in late March, although it first appeared earlier that month. It eventually disappeared as new healthy leaves grew in April. White arrows point at an instance of disorder observable in the image.

The field rover was capable of capturing growth conditions, the difference among cultivars, and some other important events during plant growth. The top and oblique cameras were capable of capturing the plants and their growth conditions (Fig. 4A–4C). The ground sampling distance of the top camera was 0.08 cm with the camera placed approximately 2.4 m above ground. The heading of wheat were clearly observed (Fig. 4D), meaning these important events could effectively be observed using our field rover. After discovering the physiological disorder on March 17, we were able to follow it backwards in time (Fig. 5) and identify that the symptom of the disorder first appeared earlier, between March 8 to 11.

**Fig. 6.**
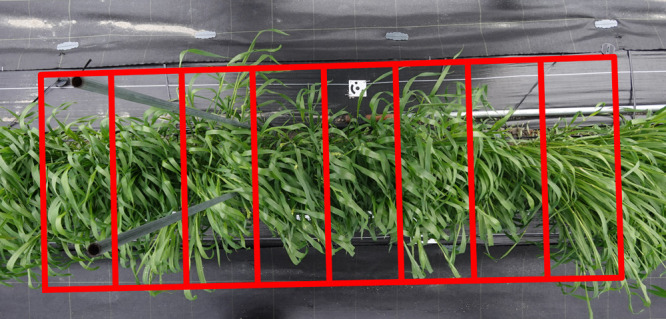
Example of automatic plot extraction using EasyIDP. Red lines denote a border between adjacent plots.

We processed those images to extract individual plots using PIX4Dmapper and EasyIDP, which allowed us to skip laborious manual plot extraction. Once images were 3D-reconstructed by using PIX4Dmapper, EasyIDP automatically extracted plots from original images for subsequent image analyses (Fig. 6).

Then, to demonstrate the phenotyping capability of the field rover, we conducted quantitative analyses of heading progression using deep learning. YOLOv5, a widely used object detection model was used. First, the deep learning models were trained using images obtained in the previous season in 2020. YOLOv5 was trained to detect wheat heads for 30 epochs and reached mAP_0.5_ value of 0.84 for the task.

**Fig. 7.**
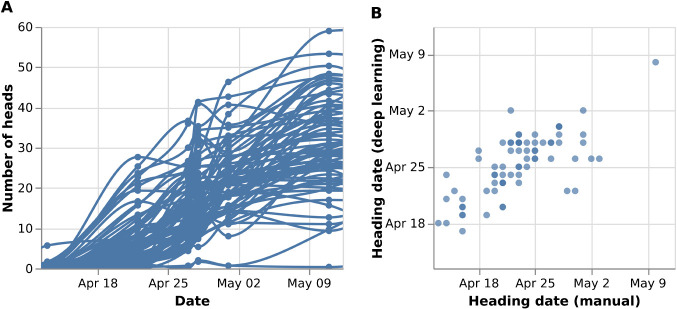
Quantitative analysis on the progression of heading using deep learning models. (A) Number of heads detected by YOLOv5. (B) Comparison of heading dates between field manual observation and image analysis.

We used those trained models for inference and it was able to identify the heading event at different timings among cultivars. Specifically, 72 plots from one furrow were used as representative for this validation and their heading progression was compared with manual observation. The number of heads detected for each plot showed an increasing curve (Fig. 7A). They surpassed the 50% threshold 5.4 days after field observation of the first head manually in the same plot (Fig. 7B). Mean absolute error of was 2.7, which was significantly smaller than randomly shuffled data (*p* < 0.01; permutation test), and the correlation coefficient was 0.72.

**Fig. 8.**
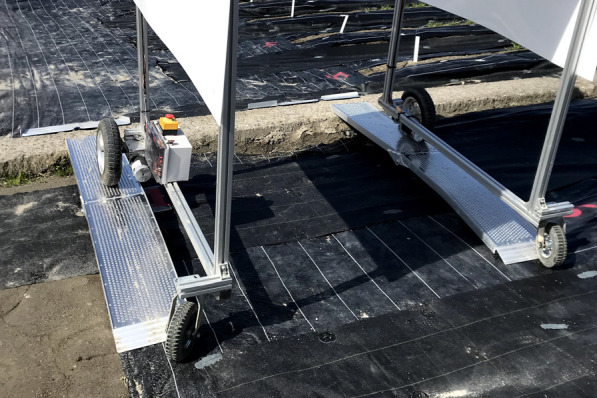
Test on slope climbing capability. The ridge between fields were roughly 30 cm higher than the field surface level. The slope was approximately 2 m long, making the slope gradient 15%. The slopes were made of aluminum and easily carried by one person.

For each phenotyping operation, the field rover had to climb a slope several times. This was because the experiment field was originally used as a rice field and surrounded by ridges and waterways. The ridges were 30 cm higher at maximum from the ground level of the field. Commercially available aluminum slope for wheelchair (Changzhou Ruedamann Tech Co., Ltd., Changzhou, China) was used for connecting two fields over the ridge. It was approximately 2 m long, making the slope gradient 15%. The field rover was capable of climbing and descending the slope smoothly (Fig. 8).

The data presented in this study are available at our GitHub repository: https://github.com/UTokyo-FieldPhenomics-Lab/UGVPP.

## Discussion

In this study, we developed a field phenotyping rover for sensing a size-limited breeding field as open-source hardware. To address the increasing demand for improving the efficiency of plant breeding, it is essential to improve the throughput and the accuracy of phenotyping. Although we conducted a trial operation at one site for wheat, the design of the field phenotyping rover is not particularly specialized to the operation and could robustly serve for a wide range of purposes in diverse field conditions and for various crop species. In contrast to UAVs, the ground-based field rover allows virtually any modification such as loading new sensors, powerful computers, and various other components for novel phenotyping applications without safety and regulatory concerns.

Our field rover was able to acquire a rich amount of image data of growing wheat. It means a wide variety of phenotyping can readily be performed by applying image analysis and machine learning techniques to the collected images. For example, plant coverage area can be easily extracted from images with a machine learning model (Guo *et al.* 2017). Moreover, heading date of wheat can also be detected by using deep learning (Hasan *et al.* 2018), which was also possible in our study. For the deep learning detection of wheat spikes, a large-scale training dataset covering diversity in wheat cultivars, field condition, and image characteristics is publicly available (David *et al.* 2020, 2021). Notably, ground-based system enables shooting at close range unlike UAVs. The ground sampling distance in our operation was 0.08 cm, a high resolution where detailed phenotypes such as presence of flowers and awns (Fig. 4D) were observable, at the same time achieving high throughput of more than 70 plots in two minutes, or more than a thousand plots per hour even including the operation to move among multiple fields. Because there is no strict limit on payload, cameras with a larger sensor size or a hyperspectral sensor could also be mounted to capture various other phenotypes. It is also possible to further expand real-time image analysis with edge-computing on the on-board computer. In fact, Jetson AGX Xavier computer can run inference with various deep learning models in real-time (Ulker *et al.* 2020). This high computational potential could also be utilized for better crop management. For example, a plant pathogen detection model could immediately send a warning when an issue arises with the plant conditions, prior to an image analysis workflow at the lab.

The remaining challenges include further automation of phenotyping operations. Our current implementation requires placing a considerable number of markers on the field. However, they could be integrated to plot labels often necessary for crop management purposes as well. We envision a more sophisticated autopilot function could achieve further improved working efficiency. Employing a hybrid approach of ground markers and a high-accuracy satellite navigation system such as real-time kinematic positioning (RTK), the field rover may be able to not only follow each furrow in a straight line but cover all furrows in a field without human intervention during the phenotyping process. Note that RTK-GPS alone would unlikely be enough to address this issue, as we also tried such a variant of our rover previously only to fail, because the signal reception was almost always unstable, presumably due to the urban surroundings with electronic interference. Even when the signal was stable, the delay in the update of coordinates made the navigation system ineffective to correct external disturbances such as small bumps. These empirical insights suggest that the hybrid approach of markers and RTK-GPS would be needed for reliable and sophisticated autopilot.

In addition, improving camera control mechanism can also help achieve making phenotyping operation effortless. The field rover currently requires manual operation to start and the stop interval shooting of each camera, which is not the most efficient method. We expect it can be controlled centrally by connecting the cameras to the main computer and using application programming interface (API) of the cameras. By developing and releasing this field rover as open-source hardware, it is expected that various researchers and engineers will be able to conduct such further development in the future.

## Author Contribution Statement

K.K. carried out experiments, analyzed the data, and drafted the manuscript with input of all authors. W.G. and K.Y. designed the methodology and crafted the hardware. S.N. conducted field experiments. K.K.S., T.T. and H.I. acquired funding and supervised the project. All authors read and approved the final manuscript.

## Figures and Tables

**Table 1.** The list of components to assemble our field rover

Component	Model name or Item number if available	Supplier	Quantity
Frame			
2200 mm	HFS8-4545-2200	MISUMI	4
1500 mm	HFS8-4545-1500	MISUMI	11
200 mm	HFS8-4545-200	MISUMI	2
90 mm	HFS8-4545-90	MISUMI	2
550 mm (slanted)	HFS8-4545-550-LCT45-RCT45	MISUMI	4
Joint bracket	HBLFSN8-45-SET	MISUMI	56
Cover film	Shiro Shiro Coat 5	C.I. Takiron	1
Drive train			
Front wheel	8inch Generic	Generic	2
Front tire	Wheelbarrow tire 3.25-8	Shinko	2
Gearbox motor	EC82M244730ALG0R	Motion Technology Electric & Machinery	2
Rear caster	JAN4944825544249	YAHATA NEJI	2
Rear tire	2.50-4HL	Tosei Sharyo	2
Electrics			
Computer	Jetson AGX Xavier	NVIDIA	1
Controller	Hex Cube Black	ProfiCNC	1
Motor driver	H-bridge 2ch 90A	AttracLab	2
Receiver	FMR-02	Futaba	1
Transmitter	SG14	Futaba	1
Battery	TB48D	SZ DJI Technology	2 or 3
DC-DC converter	Generic buck converter to 12 V and 5 V	Generic	2
Autopilot camera	Realsense D435	Intel	1
Autopilot camera mount	Multi-function Super Clamp with Double Ball Heads & 1/4ʺ Screw 1138	Smallrig	1
Phenotyping sensors*^a^*			
Camera	RX0 II	Sony	3
Mount	PLT-M6M4	LabRomance	3
Quick release adapter	QRA-635L II	Velbon	3
Misc.			
Plastic box	BCAP203018G	Takachi Electronics Enclosure	2
Emergency button	Generic	Generic	1

*^a^* Configurations for our trial; they can be changed very flexibly.
